# Promoter hypermethylation of the *Chfr* gene in neoplastic and non-neoplastic gastric epithelia

**DOI:** 10.1038/sj.bjc.6601849

**Published:** 2004-04-27

**Authors:** T Honda, G Tamura, T Waki, S Kawata, S Nishizuka, T Motoyama

**Affiliations:** 1 Department of Pathology, Yamagata University School of Medicine, 2-2-2 Iida-nishi, Yamagata 990-9585, Japan; 2 Internal Medicine, Yamagata University School of Medicine, 2-2-2 Iida-nishi, Yamagata 990-9585, Japan; 3 Laboratory of Molecular Pharmacology, National Cancer Institute, National Institute of Health, 9000 Rockville Pike, Bethesda, MD 20892, USA

**Keywords:** *Chfr*, hypermethylation, gastric cancer

## Abstract

While chromosomal instability is a common feature of human solid tumours, no abnormalities in genes involved in the mitotic checkpoint have been identified. However, recently, *Chfr* (checkpoint with forkhead associated and ring finger), a mitotic stress checkpoint gene, has been reported to be inactivated due to promoter hypermethylation in several types of human malignancy. To clarify whether *Chfr* promoter hypermethylation is involved in gastric carcinogenesis, we investigated the promoter methylation status of the *Chfr* gene in gastric cancer cell lines and primary gastric cancers. Non-neoplastic gastric epithelia from cancer-bearing and noncancer-bearing stomachs were also examined for *Chfr* promoter hypermethylation to study its cancer specificity. Two of 10 gastric cancer cell lines (20%) showed *Chfr* promoter hypermethylation with resultant loss of expression, which could be restored by 5-aza-2′ deoxycytidine treatment. *Chfr* promoter hypermethylation was present in 35% (25 of 71) of primary tumours and occurred at similar frequencies in early and advanced stages. As for non-neoplastic gastric epithelia, 1% (one of 91) from noncancer-bearing and 5% (four of 71) from cancer-bearing stomachs exhibited *Chfr* promoter hypermethylation. Thus, *Chfr* promoter hypermethylation is mostly cancer specific and frequently leads to chromosome instability in gastric cancer.

Chromosomal instability (CIN) is commonly observed in human solid tumours, with the apparent gain or loss of large parts or whole chromosomes, leading to DNA aneuploidy (Lengauer *et al*, 1997; Duesberg *et al*, 1999). In previous studies, CIN has been associated in some cases with alterations in the cell-cycle checkpoint that monitors the integrity of the spindle apparatus, a structure critical for proper bipolar segregation of duplicated sister chromatids at mitosis (Cahill *et al*, 1999). A small fraction of CIN cancers are associated with dominant mutations in the human homologues of yeast spindle checkpoint genes *BUB1* (Cahill *et al*, 1998; Imai *et al*, 1999; Gemma *et al*, 2000) and *MAD2* (Li and Benezra, 1996; Cahill *et al*, 1999). However, *BUB1* and *MAD2* mutations are relatively rare, and gastric cancers frequently exhibit DNA aneuploidy (Abad *et al*, 1998; Esteban *et al*, 1999; Imai *et al*, 1999; Russo *et al*, 2000; Tanaka *et al*, 2001).

Recently, the *Chfr* (checkpoint with forkhead associated (FHA) and ring finger (RF)) gene, involved in the mitotic stress checkpoint, was cloned and located to chromosome 12q24.33. Its product, CHFR, mediates the delayed entry into metaphase characterised microscopically by delayed chromosomal condensation (Scolnick and Halazonetis, 2000). In addition, CHFR promotes cell survival in response to mitotic stress (Scolnick and Halazonetis, 2000). CHFR possesses an N-terminal FHA domain, a central RF domain and a C-terminal cysteine-rich (CR) region (Scolnick and Halazonetis, 2000). Based on functional analysis of *Chfr* deletion mutants, both the FHA and CR regions are required for its checkpoint function. CHFR also has ubiquitin ligase activity dependent on the RF domain (Chaturvedi *et al*, 2002). Northern blot analysis of *Chfr* using RNA from eight colon, osteosarcoma and neuroblastoma cancer cell lines revealed that *Chfr* expression was absent in three cell lines (Scolnick and Halazonetis, 2000). Loss of *Chfr* expression due to hypermethylation of a CpG island in the promoter region has been observed in tumour cell lines and primary cancers of the lung, oesophagus and colon (Mizuno *et al*, 2002; Shibata *et al*, 2002; Corn *et al*, 2003; Toyota *et al*, 2003). Thus, it is possible that *Chfr* promoter hypermethylation is also involved in gastric carcinogenesis.

As promoter hypermethylation of tumour suppressor or tumour-related genes are not always cancer specific, the significance of promoter methylation status can vary among different genes (Waki *et al*, 2003a, 2003b). In the present study, we investigated *Chfr* promoter methylation status in gastric cancer cell lines, primary gastric cancers and corresponding non-neoplastic gastric epithelia, as well as in non-neoplastic gastric epithelia of noncancer-bearing stomachs to clarify both the significance and cancer specificity of *Chfr* promoter hypermethylation in gastric carcinogenesis.

## MATERIALS AND METHODS

### Gastric cancer cell lines

In all, 10 gastric cancer cell lines with variable histologies were used in our study and were cultured under appropriate conditions in our laboratory: MKN1, an adenosquamous cell carcinoma; MKN7, a well-differentiated adenocarcinoma; MKN28 and MKN74, moderately differentiated adenocarcinomas; MKN45 and KWS-I, poorly differentiated adenocarcinomas; KATO-III, a signet-ring cell carcinoma; TSG11, a hepatoid carcinoma; and ECC10 and ECC12, endocrine cell carcinomas.

### Primary gastric cancers

In all, 71 pairs of gastric cancers (40 differentiated and 31 undifferentiated carcinomas; 15 early stage and 56 advanced stage) and corresponding non-neoplastic gastric mucosa were surgically obtained from 71 patients. Tissue samples were immediately frozen and stored at −80°C until analysis. All patients received a median of 36.7 months of follow-up care (range, 1–77 months).

### Autopsy samples

Non-neoplastic gastric mucosa samples from noncancer-bearing stomachs were obtained from 34 autopsies. The autopsies consisted of 21 males and 13 females, ranging in age from 0.7 to 87 years (mean, 56 years). For most autopsies, tissue samples were obtained from the upper, middle and lower portions of the stomach. A total of 91 specimens were obtained, frozen and stored at −80°C until analysis.

### DNA and RNA extraction

DNA was extracted from the 10 gastric carcinoma cell lines, 71 primary gastric cancers and their corresponding non-neoplastic gastric mucosa, and 91 non-neoplastic gastric mucosa from autopsies using SepaGene (Sanko-Junyaku, Tokyo, Japan). Total RNA was isolated from the 10 gastric carcinoma cell lines using TRIZOL reagent (Gibco BRL, Life Technologies, Gaithersburg, MD, USA).

### Bisulfite modification and methylation-specific polymerase chain reaction (MSP)

Sodium bisulphite treatment of DNA converts all unmethylated cytosines to uracils, but leaves methylated cytosines unaffected. Briefly, 2 *μ*g aliquots of genomic DNA were denatured with sodium hydroxide and modified by sodium bisulphite. Samples were then purified using Wizard DNA purification resin (Promega, Madison, WI, USA), treated with NaOH, recovered in ethanol and resuspended in 30 *μ*l distilled water. Amplification was carried out in a 20 *μ*l reaction volume containing 2 *μ*l GeneAmp PCR Gold Buffer (PE Applied Biosystems, Foster City, CA, USA), 1.0 mM MgCl_2_, 1 *μ*l each primer, 0.2 mM dNTPs and 1 U *Taq* polymerase (AmpliTaq Gold DNA Polymerase, PE Applied Biosystems). After heating at 94°C for 10 min, PCR was performed in a thermal cycler (GeneAmp 2400, PE Applied Biosystems) for 35 cycles of denaturation at 94°C for 30 s, annealing at 54°C for 60 s and extension at 72°C for 60 s, followed by a final 7-min extension at 72°C. A positive control (Sss-I methylase-treated DNA) and negative control (distilled water without DNA) were included in each amplification. The PCR products were separated on 6% nondenaturing polyacrylamide gels. The following primer sets were used: Chfr M forward (5′-GTA ATG TTT TTT GAT AGC GGC-3′) and Chfr M reverse (5′-AAT CCC CCT TCG CCG-3′) for methylated *Chfr* sequences; Chfr U forward (5′-GGT TGT AAT GTT TTT TGA TAG TGG T-3′) and Chfr U reverse (5′-CAA ATC CCC CTT CAC CA-3′) for unmethylated *Chfr* sequences (Corn *et al*, 2003).

### Reverse transcription–PCR (RT–PCR)

Isolated RNA was reverse transcribed and amplified using the One-Step RT–PCR System (Gibco BRL). Primer sequences used were: *Chfr* forward (5′-TGG AAC AGT GAT TAA CAA GC-3′) and *Chfr* reverse (5′-AGG TAT CTT TGG TCC CAT GG-3′) for *Chfr*; and *β*-actin forward (5′-AAA TCT GGC ACC ACA CCT T-3′) and *β*-actin reverse (5′-AGC ACT GTG TTG GCG TAC AG-3′) for *β*-actin. RT–PCR products were separated on 3% agarose gels.

### 5-aza-2′-deoxycytidine (5-aza-dC) treatment

To examine the restoration of *Chfr* expression, two cell lines (MKN1 and KATO-III) were incubated for 96 h with 0.2 or 1 *μ*M 5-aza-dC (Sigma), and then harvested for RNA extraction and RT–PCR.

### Preparation of MSP-positive control

Sss-I methylase (New England BioLabs, Inc., Beverly, MA, USA) was used to methylate 100 *μ*g peripheral blood DNA, which was modified by sodium bisulphite as described above.

### Statistical analysis

Statistical comparisons were performed using Fisher's exact test, with *P*<0.05 considered statistically significant. Survival analysis was performed using a Kaplan–Meier curve with a log-rank test.

## RESULTS

### Hypermethylation and expression of *Chfr* in gastric cancer cell lines

**Figure 1:**
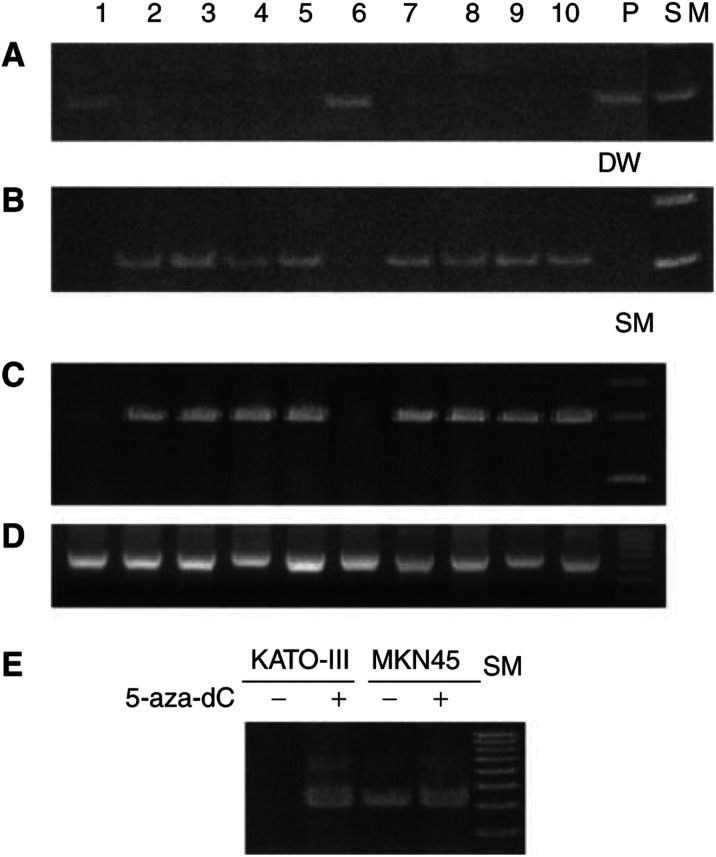
Methylation-specific polymerase chain reaction (**A** and **B**), RT–PCR (**C** and **D**) and comparison of *Chfr* mRNA expression before (−) and after (+) 5 aza-dC treatment (**E**) in gastric cancer cell lines. (**A**) *Chfr*-methylated-sequence-specific PCR and (**B**) *Chfr*-unmethylated-sequence-specific PCR. Methylated *Chfr* product is present in lanes 1 and 6 (**A**), while demethylated *Chfr* product is present in all lanes except lanes 1 and 6 (**B**). (**C**) *Chfr* RT–PCR and (**D**) *β*-actin RT–PCR (internal control). *Chfr* product is absent in lanes 1 and 6 (**C**). *β*-actin mRNA is present in all lanes (**D**). Lanes: 1, MKN1; 2, MKN7; 3, MKN28; 4, MKN45; 5, MKN74; 6, KATO-III; 7, KWS-I; 8, TSG11; 9, ECC10; 10, ECC12; P, positive control; DW, distilled water; and SM, size marker. (**E**) Treatment with 5 aza-dC restores *Chfr* mRNA expression in KATO-III, but does not affect *Chfr* expression levels in MKN45.

*Chfr* promoter hypermethylation was observed in two (MKN1 and KATO-III) of the 10 cell lines tested (Figure 1

). The remaining cells lines (MKN7, MKN28, MKN45, MKN74, KWS-I, TSG11, ECC10 and ECC12) contained unmethylated *Chfr* alleles and expressed abundant *Chfr* mRNA. MKN1 and KATO-III exhibited loss of *Chfr* expression (Figure 1), which was restored after treatment with 5-aza-dC (Figure 1). Thus, promoter methylation status of *Chfr* directly correlated with expression.

### Hypermethylation of *Chfr* in primary gastric cancers, corresponding non-neoplastic gastric mucosa and autopsy samples

**Figure 2:**
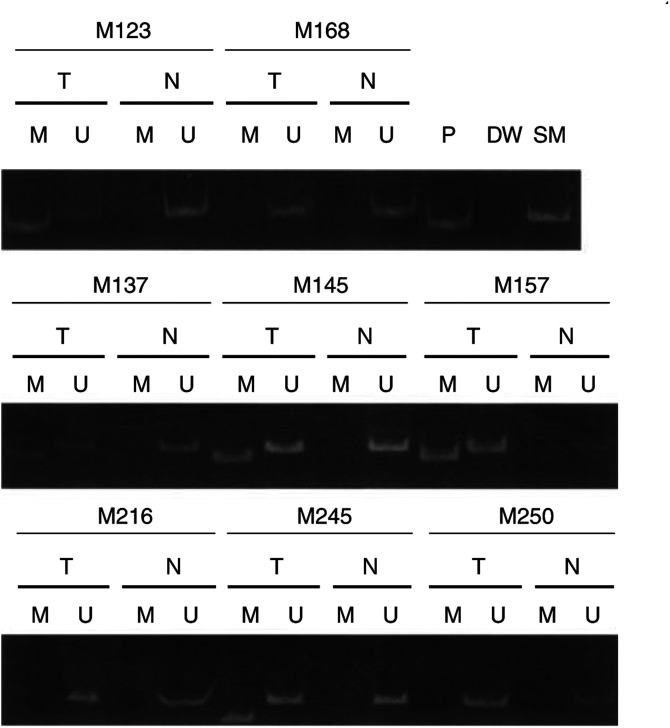
Methylation-specific polymerase chain reaction of primary gastric cancers (T) and their corresponding non-neoplastic gastric mucosa (N). M, *Chfr*-methylated-sequence-specific PCR; U, *Chf-* unmethylated-sequence-specific PCR; P, positive control; DW, distilled water; and SM, size marker. Methylated *Chfr* is present in primary gastric cancers (M123, M137, M145, M157, M245), whereas non-neoplastic gastric mucosa samples do not exhibit methylated *Chfr*.

Hypermethylation of *Chfr* was detected in 35% (25 of 71) of primary gastric cancers but only in 5% (four of 71) of the corresponding non-neoplastic gastric mucosa (Figure 2

). *Chfr* hypermethylation was observed in only one (1%) of the 91 autopsy samples. This single sample showing *Chfr* hypermethylation was obtained from the lower portion of the stomach from an 82-year-old-male patient with Parkinson's disease.

### Correlation between *Chfr* promoter hypermethylation and clinicopathological parameters

*Chfr* hypermethylation occurred at a similar frequency in early and advanced gastric cancers, and no significant correlations between *Chfr* promoter methylation status and clinicopathological factors were observed (Table 1

**Table 1:** Correlation between *Chfr* promoter methylation status and clinicopathological characteristics in gastric cancer patients

	**Promoter methylation status**		
	**Methylated**	**Unmethylated**	
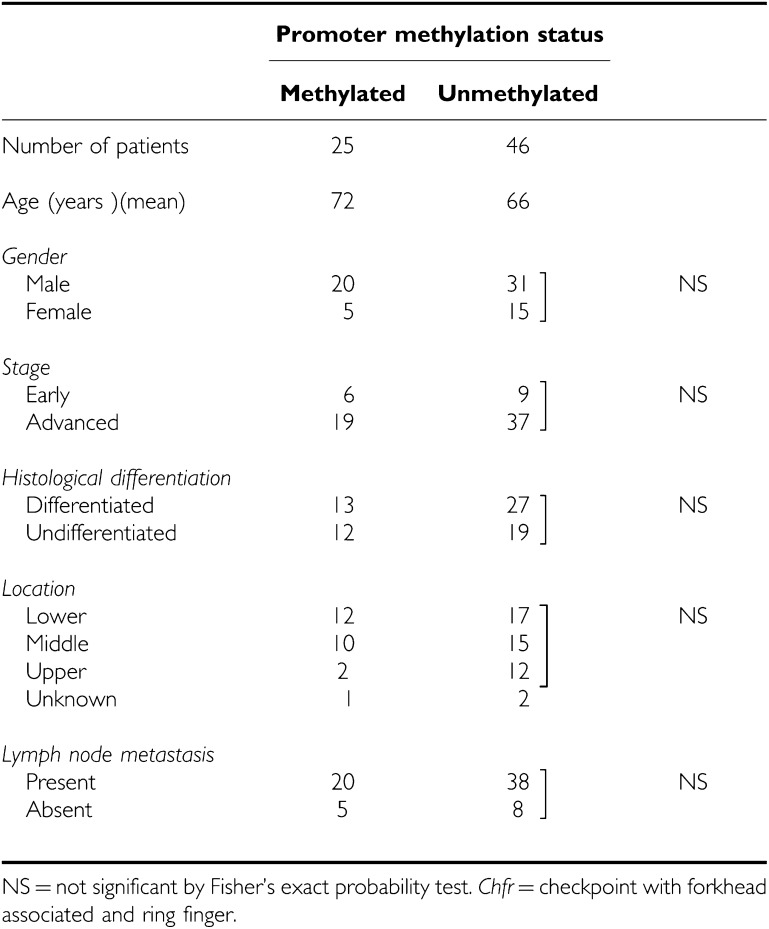			

NS=not significant by Fisher's exact probability test. *Chfr*=checkpoint with forkhead associated and ring finger.

). Methylation status did not significantly influence event-free survival rate, as analysed by Kaplan–Meier curve with log-rank test and the Breslow–Gehan–Wilcoxon test (data not shown).

## DISCUSSION

Although CIN is one of the most frequently recognised phenomenon in gastric cancer (Abad *et al*, 1998; Esteban *et al*, 1999; Russo *et al*, 2000), the mitotic checkpoint genes *hsMAD2* and *hBUB1* are rarely mutated in gastric and other types of human malignancy (Imai *et al*, 1999; Tanaka *et al*, 2001). Checkpoints upstream of the spindle checkpoint that delays chromosome condensation in response to mitotic stress are regulated by CHFR. Normal primary cells and cancer cell lines that express CHFR exhibit delayed entry into metaphase after treatment with microtubule inhibitors (Scolnick and Halazonetis, 2000). In contrast, cancer cell lines that lack CHFR enter metaphase without delay, with ectopic expression of CHFR necessary and sufficient to restore cell-cycle delay (Scolnick and Halazonetis, 2000). Recent studies of human tumours have shown that *Chfr* inactivation can be due to hypermethylation of CpGs in the promoter region (Mizuno *et al*, 2002; Shibata *et al*, 2002). However, whether *Chfr* promoter hypermethylation is involved in gastric cancer has not yet been determined.

In the present study, we showed that *Chfr* promoter hypermethylation was present in two of 10 (20%) gastric cancer cell lines and in 25 of 71 (35%) primary gastric cancers. As for non-neoplastic gastric epithelia, 5% (four of 71) of samples from cancer-bearing and 1% (one of 91) from noncancer-bearing stomachs exhibited *Chfr* promoter hypermethylation. We have shown that many tumour suppressor and tumour-related genes, such as *APC, DAP-kinase, DCC, E-cadherin, hMLH1, p16, RASSF1A* and *RUNX3*, exhibit promoter hypermethylation in both neoplastic and non-neoplastic gastric epithelia at variable frequencies (Tamura, 2004). While *GSTP1* and *PTEN* promoters remained unmethylated in both neoplastic and non-neoplastic gastric epithelia (Sato *et al*, 2002; Tamura, 2004), *TSLC1* promoter hypermethylation is highly cancer specific, but is observed at only a low frequency in gastric cancer (Honda *et al*, 2002).

Methylation generally increases with age in tissue-specific manner for different genes (Waki *et al*, 2003b). In the present study, the only sample of non-neoplastic gastric mucosa that exhibited *Chfr* hypermethylation was obtained from the noncancer-bearing stomach of an 82-year-old male patient. In contrast, *Chfr* hypermethylation was present in cancer-bearing stomachs from patients from 66 years of age. Based on these observations, it appears that age-related *Chfr* hypermethylation may constitute a general defect where individuals may become predisposed to the development of gastric cancer. The cancer specificity of hypermethylation of a particular promoter can depend on the CpG site examined (Satoh *et al*, 2002). Our present study revealed that *Chfr* promoter hypermethylation appears to be one of the most cancer-specific alterations among the various examples of tumour suppressor and tumour-related gene hypermethylation reported to date (Tamura, 2004).

While *Chfr* promoter hypermethylation is a relatively infrequent non-neoplastic gastric epithelia, it occurs at similar frequencies in early and advanced gastric cancers. This suggests that *Chfr* promoter hypermethylation may be an early event in gastric carcinogenesis. DNA aneuploidy has been observed in 50–71% of gastric cancers and correlates with poor prognosis (Abad *et al*, 1998; Esteban *et al*, 1999; Russo *et al*, 2000). In the present study, we failed to find a statistically significant correlation between *Chfr* hypermethylation and gastric cancer patient survival. Nonetheless, our results did display a tendency towards a worse prognosis in patients with tumours that displayed *Chfr* hypermethylation. Owing to the lack of a significant correlation between *Chfr* methylation status and prognosis, and the relatively low frequency of *Chfr* hypermethylation compared to that of DNA aneuploidy, other gene(s) and/or mechanism(s) are likely to also contribute to CIN in gastric cancer.

In conclusion, *Chfr* promoter hypermethylation frequently occurs as an early event of gastric carcinogenesis. Owing to its cancer specificity, detection of *Chfr* promoter methylation could be useful as a molecular diagnostic marker for gastric cancer.

## References

[bib1] Abad M, Ciudad J, Rincon MR, Silva I, Paz-Bouza JI, Lopez A, Alonso AG, Bullon A, Orfao A (1998) DNA aneuploidy by flow cytometry is an independent prognostic factor in gastric cancer. Anal Cell Pathol 16: 223–231 9762369 10.1155/1998/158243 PMC4611107

[bib3] Cahill DP, Kinzler KW, Vogelstein B, Lengauer C (1999) Genetic instability and Darwinian selection in tumor. Trends Cell Biol 9: M57–M60 10611684

[bib4] Cahill DP, Lengauer C, Yu J, Riggins GJ, Wilson JK, Markowitz SD, Kinzler KW, Vogelstein B (1998) Mutation of mitotic checkpoint genes in human cancers. Nature 392: 300–303 9521327 10.1038/32688

[bib5] Chaturvedi P, Sudakin V, Bobiak ML, Fisher PW, Mattern MR, Jablonski SA, Hurle MR, Zhu Y, Yen TJ, Zhou BBS (2002) Chfr regulates a mitotic stress pathway through its RING-finger domain with ubiquitin ligase activity. Cancer Res 62: 1797–1801 11912157

[bib6] Corn PG, Summers MK, Fogt F, Virmani AK, Gazdar AF, Halazonetis TD, Ei-Deiry WS (2003) Frequent hypermethylation of the 5′ CpG island of the mitotic stress checkpoint gene *Chfr* in colorectal and non-small cell lung cancer. Carcinogenesis 24: 47–51 12538348 10.1093/carcin/24.1.47

[bib7] Duesberg P, Ransnick D, Li R, Winters L, Rausch C, Hehlmann R (1999) How aneuploidy may cause cancer and genetic instability. Anticancer Res 19: 4887–4906 10697602

[bib9] Esteban F, Vega DS, Garcia R, Rodriguez R, Manzanares J, Tamames S (1999) DNA content by flow cytometry in gastric carcinoma: pathology, ploidy and prognosis. Hepatogastroenterology 46: 2039–2043 10430394

[bib12] Gemma A, Seike M, Seike Y, Uematsu K, Hibino S, Kurimoto F, Yoshimura A, Shibuya M, Harris CC, Kudoh S (2000) Somatic mutation of the *hBUB1* mitotic checkpoint gene in primary lung cancer. Gene Chromosomes Cancer 29: 213–218 10.1002/1098-2264(2000)9999:9999<::aid-gcc1027>3.0.co;2-g 10992296

[bib13] Honda T, Tamura G, Waki T, Jin Z, Sato K, Motoyama T, Kimuta W, Kawata S, Nishizuka S, Murakami Y (2002) Hypermethylation of *TSLC1* gene promoter in gastric cancer. Jpn J Cancer Res 93: 857–860 12716461 10.1111/j.1349-7006.2002.tb01329.x PMC5927103

[bib14] Imai Y, Shiratori Y, Kato N, Inoue T, Omata M (1999) Mutational inactivation of mitotic checkpoint genes, *hsMAD2* and *hBUB1*, is rare in sporadic digestive tract cancers. Jpn J Cancer Res 90: 837–840 10543255 10.1111/j.1349-7006.1999.tb00824.x PMC5926140

[bib20] Lengauer C, Kinzler KW, Vogelstein B (1997) Genetic instability in colorectal cancers. Nature 386: 623–627 9121588 10.1038/386623a0

[bib21] Li Y, Benezra R (1996) Identification of a human mitotic checkpoint gene: *hsMAD2*. Science 274: 246–248 8824189 10.1126/science.274.5285.246

[bib22] Mizuno K, Osada H, Konishi H, Tatematsu Y, Tatabe Y, Mitsudomi T, Fujii Y, Takahashi T (2002) Aberrant hypermethylation of the *CHFR* prophase checkpoint gene in human lung cancer. Oncogene 21: 2328–2333 11948416 10.1038/sj.onc.1205402

[bib24] Russo A, Bazan V, Migliavacca M, Zanna I, Tubiolo C, Tumminello FM, Dardanoni G, Cajozzo M, Bazan P, Modica G, Latteri M, Tomasino RM, Colucci G, Gebbia N, Lato G (2000) Prognostic significance of DNA ploidy, S-phase fraction, and tissue levels of aspartic, cysteine, and serine proteases in operable gastric carcinoma. Clin Cancer Res 6: 178–184 10656448

[bib25] Sato K, Tamura G, Tsuchiya T, Endoh Y, Sakata K, Motoyama T, Usuba O, Kimura W, Terashima M, Nishizuka S, Zou S, Meltzer SJ (2002) Analysis of genetic and epigenetic alterations of the *PTEN* gene in gastric cancer. Virchows Arch 440: 160–165 11964046 10.1007/s004280100499

[bib26] Satoh A, Toyota M, Itoh F, Kikuchi T, Obata T, Sasaki Y, Suzuki H, Yawata A, Kusano M, Fujita M, Hosokawa M, Yanagihara K, Tokino T, Imai K (2002) DNA methylation and histone deacetylation associated with silencing *DAP-kinase* gene expression in colorectal and gastric cancers. Br J Cancer 86: 817–823 10.1038/sj.bjc.6600319 PMC2375414 12087472

[bib28] Scolnick DM, Halazonetis TD (2000) *Chfr* defines a mitotic stress checkpoint that delays entry into metaphase. Nature 406: 430–435 10935642 10.1038/35019108

[bib29] Shibata Y, Haruki N, Kuwabara Y, Ishiguro H, Shinoda N, Sato A, Kimura M, Koyama H, Toyama T, Nishiwaki T, Kudo J, Terashita Y, Konishi S, Sugimura H, Fujii Y (2002) *Chfr* expression is downregulated by CpG island hypermethylation in esophageal cancer. Carcinogenesis 23: 1695–1700 12376479 10.1093/carcin/23.10.1695

[bib31] Tamura G (2004) Promoter methylation status of tumor suppressor and tumor-related genes in neoplastic and non-neoplastic gastric epithelia. Histol Histopathol 19: 221–228 14702190 10.14670/HH-19.221

[bib33] Tanaka K, Nishioka J, Kato K, Nakamura A, Mouri T, Miki C, Kusunoki M, Nobori T (2001) Mitotic checkpoint protein hsMAD2 as a marker predicting liver metastasis of human gastric cancers. Jpn J Cancer Res 92: 952–958 11572763 10.1111/j.1349-7006.2001.tb01186.x PMC5926839

[bib34] Toyota M, Sasaki Y, Satoh A, Ogi K, Kikuchi T, Suzuki H, Mita H, Tanaka N, Itoh F, Issa JP, Jair KW, Schuebel KE, Imai K, Tokino T (2003) Epigenetic inactivation of CHFR in human tumors. Proc Natl Acad Sci USA 100: 7818–7823 12810945 10.1073/pnas.1337066100 PMC164671

[bib35] Waki T, Tamura G, Sato M, Motoyama T (2003a) Age-related methylation of tumor suppressor and tumor-related genes: an analysis of autopsy samples. Oncogene 22: 4128–4133 12821947 10.1038/sj.onc.1206651

[bib36] Waki T, Tamura G, Sato M, Terashima M, Nishizuka S, Motoyama T (2003b) Promoter methylation status of *DAP-kinase* and *RUNX3* genes in neoplastic and non-neoplastic gastric epithelia. Cancer Sci 94: 360–364 12824905 10.1111/j.1349-7006.2003.tb01447.x PMC11160204

